# An Empirical Study on Supporting Work‐Life Balance for Working Carers: Analysis of the Impact of Health Status, Work Environment, and Care Environment

**DOI:** 10.1002/alz70860_104505

**Published:** 2025-12-23

**Authors:** Aya Seike, Sayaka Takeuchi, Junko Hagihara, Rieko Inokuchi, Chiharu Moriyama, Shigemi Nanpo, Keiko Hara, Yumi Shigesada, Takahiko Morinaga, Yuichiro Yano, Akinori Takeda, Takashi Sakurai, Hidenori Arai

**Affiliations:** ^1^ Faculty of Sports and Health Science, Ritsumeikan University, Kusatsu city, Shiga, Japan; ^2^ Division of Regional Development System Design, Graduate School of Agriculture, Hokkaido University, Sapporo, Hokkaido, Japan; ^3^ National Center for Geriatrics and Gerontology The Center for Comprehensive Care and Research on Memory Disorders, Ohbu, Japan; ^4^ National Center for Geriatrics and Gerontology The Center for Comprehensive Care and Research on Memory Disorders, Ohbu, Aichi, Japan; ^5^ National Center for Geriatrics and Gerontology, The Center for Comprehensive Care and Research on Memory Disorders, Ohbu, Aichi, Japan; ^6^ Faculty of Sports and Health Science, Ritsumeikan University Research Fields: Applied Health Science, Social Medicine, Gerontology and Geriatrics, Kusatsu, Shiga, Japan; ^7^ Faculty of Sports and Health Science, Ritsumeikan University, Kusatsu, Shiga, Japan; ^8^ Department of General Medicine, Juntendo University Faculty of Medicine, Tokyo, Tokyo, Japan; ^9^ Juntendo University AI Incubation Center, Tokyo, Japan; ^10^ National Center for Geriatrics and Gerontology, Obu, Aichi, Japan

## Abstract

**Background:**

In Japan, where population aging is rapidly progressing, the increase in working carers has become a pressing social issue. Recent data indicate that 106,000 people leave their jobs annually due to caring responsibilities, raising the risk of presenteeism and absenteeism. These challenges necessitate understanding how the work environment, carers' health, and care environment influence work‐life balance and productivity. This study aimed to examine these factors quantitatively and propose evidence‐based support measures to improve the well‐being and productivity of working carers.

**Method:**

A survey targeting 100 working carers at the National Center for Geriatrics and Gerontology was conducted from May to June 2024. Data were collected on basic attributes, work environment (psychological safety), caring conditions, Health‐Related Quality of Life (SF‐36), and presenteeism (Wfun scale). Structural equation modeling (SEM) was employed to evaluate the relationships between these factors and work‐life balance, focusing on psychological, physical, and social dynamics.

**Result:**

The respondents’ average age was 53.9±7.5 years (21.0% men, 79.0% women). Care recipients’ average age was 81.4±7.2 years, with 45.0% having dementia, 25.0% cerebrovascular diseases, and 30.0% other chronic illnesses. The Wfun scale showed that 66.0% could work energetically, while 19.0% experienced occasional difficulties, and 6.0% required work adjustments. SEM analysis revealed that the work environment moderately influenced work‐life balance through psychological safety and support system usage (coefficient=0.298). Carers' health had the strongest impact, mediated by physical and mental health (coefficient=0.712). The care environment showed a weaker influence, primarily through consultation systems and available services (coefficient=0.162).

**Conclusion:**

This study demonstrated that improving psychological safety and maintaining carers' physical and mental health are crucial for balancing work and caring. Promoting support systems is vital for improving the care environment and productivity. Future research will collect longitudinal data to explore early intervention points and develop assessment tools to evaluate long‐term outcomes, ensuring more sustainable support for working carers.

